# Exosomes Derived From Hypertrophic Cardiomyocytes Induce Inflammation in Macrophages *via* miR-155 Mediated MAPK Pathway

**DOI:** 10.3389/fimmu.2020.606045

**Published:** 2021-02-03

**Authors:** Hui Yu, Lei Qin, Yunzhi Peng, Wenhui Bai, Zhanli Wang

**Affiliations:** ^1^ School of Basic Medicine, Baotou Medical College, Baotou, China; ^2^ Inner Mongolia Key Laboratory of Disease-Related Biomarkers, Baotou Medical College, Baotou, China; ^3^ Genetic Eugenics Department, Inner Mongolia Maternal and Child Health Care Hospital, Huhhot, China

**Keywords:** cardiac hypertrophy, exosome, microRNA, macrophage, inflammation

## Abstract

The inflammatory immune microenvironment plays an important role in the development of cardiac hypertrophy. Exosomes have emerged as the potent modulators of inflammatory responses. This study aimed to determine how exosomes derived from angiotensin II (Ang II)-induced hypertrophic cardiomyocytes (HCs) interfere with the inflammatory signal pathways in macrophages. Herein, we showed that increased exosome release was observed in HCs when compared to normal cardiomyocytes (NCs). Incubation of the murine macrophage cell line RAW264.7 in the presence of exosomes isolated from the culture media of HCs triggers the secretion of inflammatory cytokines interleukin (IL)-6 and IL-8. Cytokines release induced by HCs-derived exosomes was prevented by down-regulation of Argonaute2 (AGO2), suggesting that the non-coding RNAs were involved in exosome-induced inflammatory responses in RAW 264.7 macrophages. RNA sequencing assays further demonstrated that a total of seven microRNAs were differentially expressed between NCs-derived and HCs-derived exosomes. Importantly, miR-155 played a crucial role in the initiation of inflammation in macrophages. Further analyses demonstrated that HCs-derived exosomes induced the phosphorylation of extracellular signal-regulated kinase (ERK), c-Jun N-terminal kinase (JNK), and p38 *via* miR-155. Our results support the concept that exosomal microRNAs have emerged as important inflammatory response modulators regulating cardiac hypertrophy.

## Introduction

Cardiac hypertrophy is commonly observed in some patients with cardiac disease such as congestive heart failure, valve disease, and hypertension (1). The dominant response of the heart to almost all forms of hemodynamic overload, myocardial injury, or endocrine disorders will ultimately lead to cardiomyocyte hypertrophy (2). It has been reported that the development of cardiac hypertrophy may be attributed to multiple signaling pathways, such as mTOR, PI3K-Akt, AMPK, and MAPK (3). In general, blocking one or some of the hypertrophic signaling pathways could alleviate the development of cardiac hypertrophy.

Despite the achievements obtained over the past decades, the pathology of cardiac hypertrophy is still poorly understood. Exosomes are 30–120 nm endocytic membrane-derived vesicles that are secreted by a variety of different cell types. It is well known that exosomes play significant roles in multiple biological processes including development, cell differentiation, and stress response (4). The investigation of exosome-mediated intercellular communication by shuttling nucleic acids, proteins, and lipids has become an important topic in studies of health and illness (5). Recently, researchers also confirmed that exosomes might act as vehicles to deliver cargos to reprogram the cardiac microenvironment, which was believed to contribute to the development of several cardiovascular diseases (6).

Most studies have focused on the characterization of exosomes isolated from cardiomyocytes (7). Previous studies have revealed that the generation and release of exosomes from cardiomyocytes are influenced by many factors, including angiotensin II (Ang II), hypoxia, inflammation, or injury (8). Additionally, the composition of exosomes is influenced by the pathophysiological status for exosomes formation, suggesting the potential functional diversity of exosomes (9). Moreover, exosomes from cardiomyocytes regulate the functions of various host cell types. This regulation is known to primarily involve the physiological and pathological roles in the heart (10). However, molecular features and functions of exosomes from hypertrophic cardiomyocytes have been poorly studied.

In the present study, we investigated if the release of exosomes from hypertrophic cardiomyocytes (HCs) can affect the pro-inflammatory signal transduction pathways in macrophages. Our results suggest that exosome-mediated transfer of miRNAs constitute a novel communication mode between cardiomyocytes and macrophages, indicating a potential role in novel treatments of cardiac hypertrophy.

## Materials and Methods

### Primary Culture of Neonatal Rat Cardiomyocytes

The hearts of 1- to 2-day-old neonatal Wistar rats were minced and incubated in a solution with the addition of 1 mg/ml collagenase II and 0.12% trypsin purchased from Sigma Aldrich Co. (St. Louis, MO, USA) at 37°C. Following 10 min incubation, the first supernatant was discarded. The following supernatants were collected from several cycles of digesting with a mixture of collagenase II and trypsin enzyme until the tissue was homogenized. In each cycle, the fetal calf serum (HyClone, Waltham, MA, USA) was added to stop homogenization. The dissociated cells were enriched for cardiomyocytes *via* differential adhesion for 60 min and were plated on a medium supplemented with 0.1 mM 5′-bromo-2′-deoxyuridine (BrdU). All animal studies were carried out in strict accordance with the recommendations in the Guide for the Care and Use of Laboratory Animals and approved by the local ethics committee.

### Angiotensin II-Induced Hypertrophy Model of Cardiomyocytes

After 24 h of serum starvation, the primary neonatal rat cardiomyocytes were treated with or without 1 μmol/L Ang II for 48 h to stimulate hypertrophy. The hypertrophic cardiomyocytes were identified by real-time quantitative PCR (qRT-PCR) assay. Total RNA was extracted from the hypertrophic cardiomyocytes using the TRIzol from Invitrogen (Carlsbad, CA, USA). Complementary DNA (cDNA) was obtained using the PrimeScript RT Master Mix Kit (Takara, Japan). A SYBR Premix Ex Taq II Kit (Takara, Japan) was used to perform the qRT-PCR assay. The following primers (Sangon Biotech, Shanghai, China) were applied: ANP: 5′-CTC CCA GGC CAT ATT GGAG-3′ (sense) and 5′-TCC AGG TGG TCT AGC AGGTT -3′ (anti-sense); BNP: 5′-TGG GAA GTC CTA GCC AGT CTC-3′ (sense) and 5′-TCT GAG CCA TTT CCT CTGAC-3′ (anti-sense); GAPDH: 5′-GGC ACA GTC AAG GCT GAG AATG-3′ (sense) and 5′-ATG GTG GTG AAG ACG CCA GTA-3′ (anti-sense).

### Exosomes Extraction and Validation

Exosomes in the cardiomyocyte culture medium were isolated using total exosome isolation reagent from Invitrogen (Carlsbad, CA, USA). The presence of exosomes was confirmed using H-7650 scanning electron microscopy (SEM; Hitachi, Japan). For SEM assay, exosomes were placed on copper grids, stained with uranyl acetate, and examined. Analysis of exosome sizes was done using the SEM images *via* ImageJ. Besides, western blot was performed to verify the presence of exosomes with primary antibodies as described previously (11). Moreover, acetylcholinesterase (AChE) activity was used to quantify the level of exosomes. AChE assay was performed with the Amplex Red Acetylcholine/Acetylcholinesterase Assay Kit (Thermo Fisher Scientific, CA, USA) according to the manufacturer’s instructions.

### Culture and Treatment of RAW264.7 Cells

The murine macrophage cell line RAW264.7 was purchased from the Cell Bank of the Chinese Academy of Medical Sciences (Beijing, China). The cells were maintained in Dulbecco’s modified Eagle’s medium (DEME) containing 10% fetal bovine serum and supplemented with 100 unit/ml penicillin and 100 μg/ml of streptomycin (Solarbio, Beijing, China) in a humidified incubator at 37°C containing 5% CO_2_. For the treatment of macrophages with cardiomyocyte-derived exosomes, RAW264.7 cells were seeded in 6-well plates at a mean of 3×10^5^ cells/well, and the extracted exosomes were added to the culture medium to a final concentration of 100 μg/ml.

### Cytokine Assays

The cell supernatant was collected and centrifuged at 10,000×g for 15 min at 4°C to remove cell debris. The secretion levels of pro-inflammatory factors including IL-6 and IL-8 were measured in duplicate using ELISA kits following the manufacturer’s instructions (Solarbio, Beijing, China). Absorbance was then measured at 450 nm.

### Cell Transfection

RAW264.7 macrophages were seeded in 6-well plates and transfected using the siPORT NeoX transfection reagent (Ambion, Austin, TX, USA) according to the manufacturer’s instructions. AGO2-specific siRNA and a scrambled negative control were purchased from Ambion (Austin, TX, USA). Additionally, miR-155 mimic, mimic control, miR-155 inhibitor, and inhibitor control were obtained from Ambion (Austin, TX, USA). After 48 h of transfection, cells were harvested for analysis.

### RNA Isolation, Sequencing, and Data Processing

Total exosomal RNA was extracted with TRIzol from Invitrogen (Carlsbad, CA, USA). The Illumina TrueSeq small RNA library prep kit (San Diego CA, USA) was then used to construct a small RNA library from exosomal RNA obtained. Adapters were directly, and specifically, ligated to the 3′ and 5′ end of the RNA. The ligated RNA molecule was reverse transcribed into single-stranded cDNA for PCR amplification. After that, sequencing was then performed on an Illumina HiSeq 2000 sequencer (San Diego, CA, USA).

### Target Gene Prediction and Function Enrichment Analysis

The putative targets of miRNAs were predicted by the softwares TargetScan, miRmap, PITA, PicTar, microT, miRanda, and RNA22. Gene Ontology (GO) annotation was then used to classify the functions of the predicted genes. Further Kyoto Encyclopedia of Genes and Genomes (KEGG) analysis was performed with these predicted genes. Fisher’s exact test was used to determine the significance of the GO terms and pathways, and the false discovery rate (FDR) was calculated to correct the p-value. A corrected p-value below 0.05 was considered statistically significant.

### Validation of miRNAs

Expression ratios of differentially expressed miRNAs obtained by sequencing were further validated by qRT-PCR analysis. Primers for qRT-PCR were purchased from Applied Biosystems. The expression of miRNAs was normalized to the level of U6 small nuclear RNA (snRNA). The experiments were carried out in triplicate. The relative expression would be performed using the 2^−ΔΔCt^ method, with U6 snRNA used as an endogenous reference.

### Western Blot Analysis

For western blotting analysis, the whole-cell lysates were extracted with a protein extraction kit (Beyotime, Shanghai, China). Equal amounts of protein were separated by 10% sodium dodecyl sulfate-polyacrylamide gel electrophoresis (SDS-PAGE) and transferred to polyvinylidene difluoride (PVDF) membranes. The membranes were blocked with 5% skim milk in a TBS buffer for 1 h and then incubated with primary antibodies overnight at 4°C, including p38, phosphorylated p38, ERK1/2, phosphorylated ERK1/2, JNK, phosphorylated JNK, AGO2, CD63, and CD81 (1:1,000 dilution; Cell Signaling, Danvers, MA, USA). The membrane was washed with TBS buffer and incubated with peroxidase-conjugated anti-mouse or anti-rabbit secondary antibodies (1:500, Proteintech, Chicago, USA) for 1 h. The western blot bands were detected with an enhanced chemiluminescence reagent (Solarbio, Beijing, China) and were measured using ImageJ software. GAPDH or alpha-tubulin served as the internal reference.

### Statistical Analysis

Statistical analysis was performed using SPSS software (version 20.0; IBM, USA). Data comparisons between two groups were performed using the Student’s t-test. The differences were considered significant when the p-value was below 0.05.

## Results

### Angiotensin II Induces Cardiomyocyte Hypertrophy

**Figure 1:**
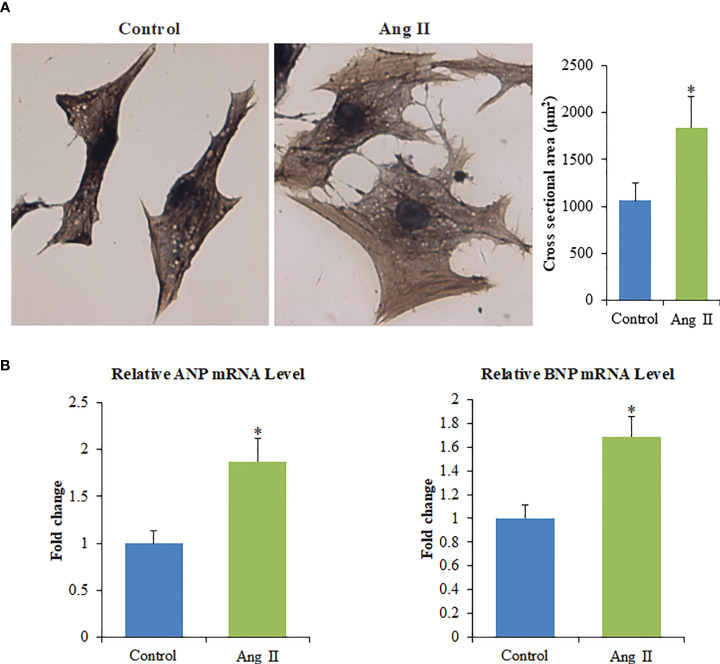
Ang II induces the hypertrophy of primary cardiomyocytes *in vitro*. **(A)** Left panel: The cell surface of cardiomyocytes quantified with ImagePro-Plus 6.0, Scale bar = 100 µm; Right panel: Cross-sectional area of cardiomyocytes. **(B)** The mRNA levels of ANP and BNP. Values are represented as mean ± SD from three independent determinations. *p < 0.05, *vs* control group.

The cardiomyocyte hypertrophy model was established. Ang II-mediated enlargement of cardiomyocytes as evidenced by increased cell surface area (**Figure 1A**). The expressions of atrial natriuretic peptide (ANP) and B-type natriuretic peptide (BNP) (**Figure 1B**) of the Ang II group were appreciably higher than those of the control group (p<0.05). These results show that the cardiomyocyte hypertrophy model was successfully established.

### Identification of Cardiomyocyte-Derived Exosomes

**Figure 2:**
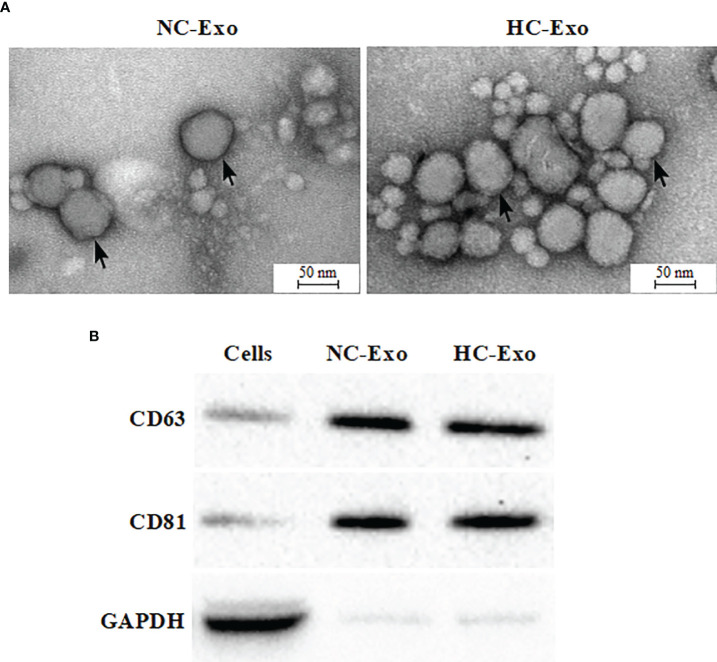
Characterization of exosomes derived from hypertrophic cardiomyocytes. **(A)** Electron micrograph of normal cardiomyocyte-derived exosomes (left) and hypertrophic cardiomyocyte-derived exosomes (right). **(B)** Western blot analysis of exosome biomarkers CD63 and CD 81. Immunoblots are representative of three separate experiments.

Exosomes were isolated according to the manufacturer’s protocol. Either normal cardiomyocyte-derived exosomes (NC-Exo) or hypertrophic cardiomyocyte-derived exosomes (HC-Exo) were validated. SEM assay implied that exosomes from two groups exhibited a round morphology and uniform distribution in diameter of about 50–100 nm (**Figure 2A**). In contrast to exosomes, apoptotic vesicles and necrotic bodies displayed irregular shapes and heterogeneous size distribution. Western blot analysis showed that the expression levels of the exosomal marker proteins CD63 and CD81 were enriched (**Figure 2B**). These results indicated the successful isolation of exosomes. We further measured AChE activity to determine the effect of Ang II treatment on exosome release. As shown in Support Information **Figure S1**, Ang II treatment significantly increased AChE activity compared to the control group, suggesting that Ang II treatment stimulates exosome secretion from hypertrophic cardiomyocytes.

### Cardiomyocyte-Derived Exosomes Affect the Production of Cytokines

**Figure 3:**
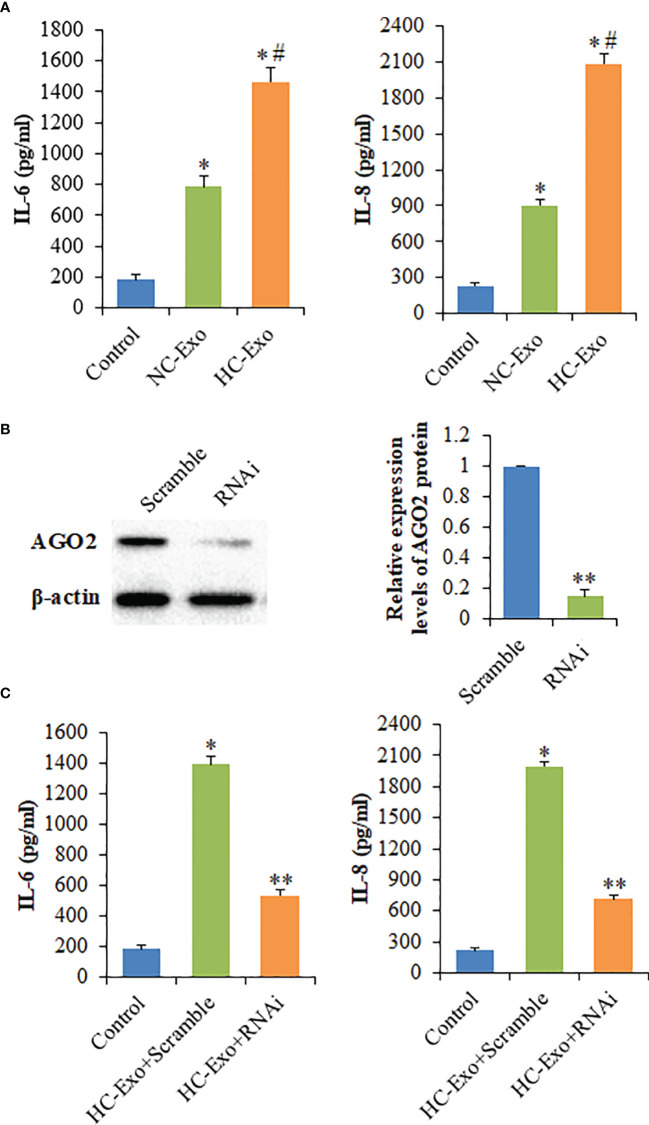
The effects of cardiomyocyte-derived exosomes on the production of cytokines. **(A)** The levels of IL-6 and IL-8 secreted by macrophages following the treatment with cardiomyocyte-derived exosomes. **(B)** Left panel: Western blot analysis of AGO2 protein following the treatment with AGO2 siRNA; Right panel: Densitometry analysis of AGO2 protein levels. **(C)** The levels of IL-6 and IL-8 secreted by exosome-treated macrophages following the treatment with AGO2 siRNA. Values are represented as mean ± SD from three independent determinations. *p < 0.05, *vs* control group. ^#^p < 0.05, *vs* NC-Exo group. **p < 0.05, *vs* Scramble group.

Compared with the NC-Exo, HC-Exo significantly increased IL-6 and IL-8 production of RAW264.7 cells (**Figure 3A**). To rule out the possibility that the elevation in cytokine levels was due to HC-Exo or the supernatant of hypertrophic cardiomyocytes, the levels of IL-6 and IL-8 in HC-Exo, and the supernatant of hypertrophic cardiomyocytes were determined. The results showed that the contents of IL-6 and IL-8 were not detected. Taken together, these data suggested that HC-Exo mediated the immune-inflammatory responses of macrophages. To determine whether cytokines release induced by HC-Exo was influenced by AGO2, the active siRNA was used to knockdown AGO2 expression (**Figure 3B**). Compared with the scrambled negative control group, the significantly decreased production of IL-6 and IL-8 was observed in the siRNA group (**Figure 3C**), indicating that exosome-mediated transfer of the non-coding RNAs might involve in the cytokines secretion by RAW264.7 cells.

### The miRNAs Were Differentially Expressed in Hypertrophic Cardiomyocyte Exosomes

**Figure 4:**
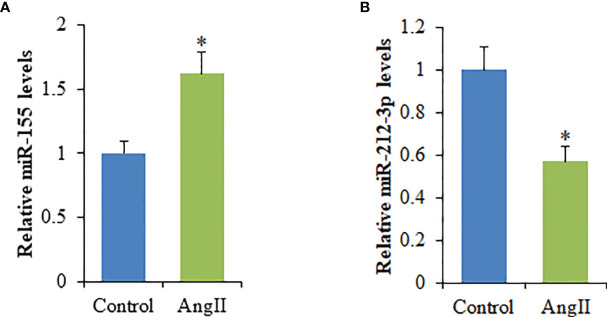
qRT-PCR validation of the dysregulated expression of selected miRNAs in cardiomyocyte-derived exosomes. **(A)** miR-155. **(B)** miR-212-3p. Values are represented as mean ± SD from three independent determinations. *p < 0.05, vs control group.

The miRNA expression profiles in cardiomyocyte-derived exosomes were determined by high throughput miRNA sequencing, and 635 differentially expressed miRNAs were found. However, compared to NC-Exo, a total of seven differentially expressed miRNAs were identified in HC-Exo. Among these, four miRNAs were upregulated while three were downregulated in HC-Exo compared with NC-Exo (p<0.05). **Table 1** demonstrated seven differentially expressed miRNAs in each group. We used qRT-PCR to characterize relative expression levels of miR-155 and miR-212-3p to validate the data obtained through miRNA sequencing. The results obtained through qRT-PCR and miRNA sequencing were essentially identical (**Figure 4**).

**Table 1:** The differentially expressed microRNAs (miRNAs) in exosomes derived from normal and hypertrophic cardiomyocytes.

miRNA ID	Count_Control	Count_AngII	Fold change	Up/down	P-value
miR-155	6.146	28.313	1.896987329	Up	0.000707545
miR-206-3p	14.34	55.649	1.649553117	Up	0.000016341
miR-199a-5p	19.461	57.602	1.258775438	Up	0.000342907
miR-494-3p	31.753	83.962	1.096086007	Up	0.000107917
miR-212-3p	52.238	30.265	−1.094206933	Down	0.000694148
let-7f-1-3p	59.408	34.171	−1.104642374	Down	0.000267227
miR-30c-2-3p	13.316	1.953	−3.076154543	Down	0.000639087

### Functional Annotation and Enrichment Analysis of Differentially Expressed miRNAs

**Figure 5:**
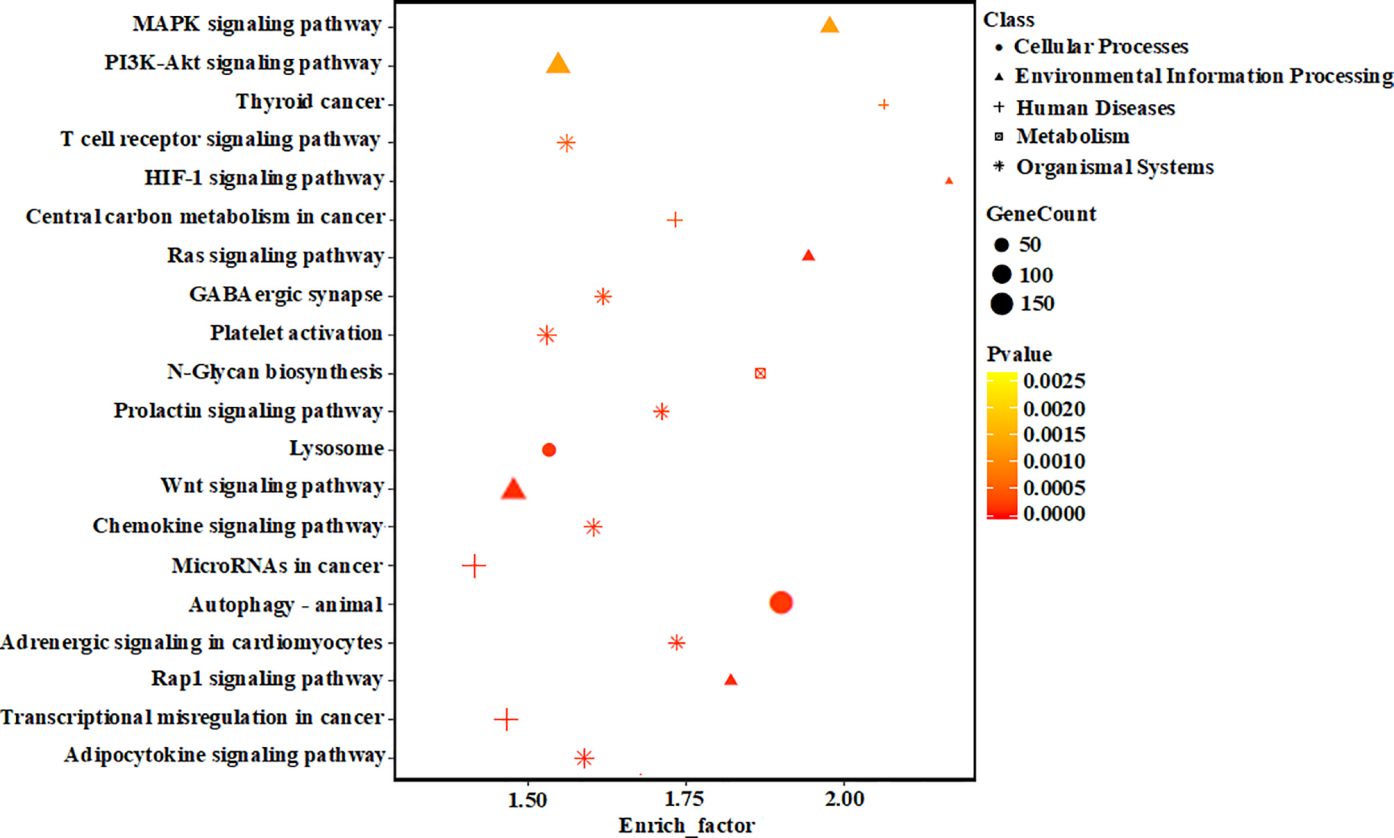
KEGG pathway analyses of the target genes of the differentially expressed miRNAs. The vertical axis represents the pathway name. The horizontal axis represents the enrich factor: enrich factor = (dysregulated gene number in a pathway/total dysregulated gene number)/(gene number in a pathway in the database/total gene number in the database). The top 20 pathway terms were selected according to the enrich factor value. Different colors from yellow to red represent p-value, and the size of the dot represents the gene count number in a pathway.

To elucidate the potential role of miRNAs in hypertrophic cardiomyocytes, the prediction of miRNA targets was performed and 11,637 genes were obtained. To reduce the false positives rate of target gene prediction, only the predicted targets within the three databases described above were further analyzed. Finally, 5,477 predicted target genes were selected for further investigation. As shown in support information **Figure S2**, the differentially expressed miRNAs were significantly enriched in various biological process, including the categories of immune system process (GO: 0002376), cell morphogenesis (GO: 0000902), regulation of protein phosphorylation (GO: 0001932), positive regulation of protein phosphorylation (GO: 0001934), and immune system development (GO: 0002520), respectively. Target genes were mapped to KEGG pathways, and 136 pathways in “hypertrophic *vs*. normal” cardiomyocytes exosomes were enriched. The top 20 KEGG pathways significantly enriched in hypertrophic cardiomyocytes exosomes were shown in **Figure 5**, including the MAPK signaling pathway and PI3K-Akt signaling pathway.

### MiR-155 Is Transferred by Exosomes and Regulates Macrophage Activation

**Figure 6:**
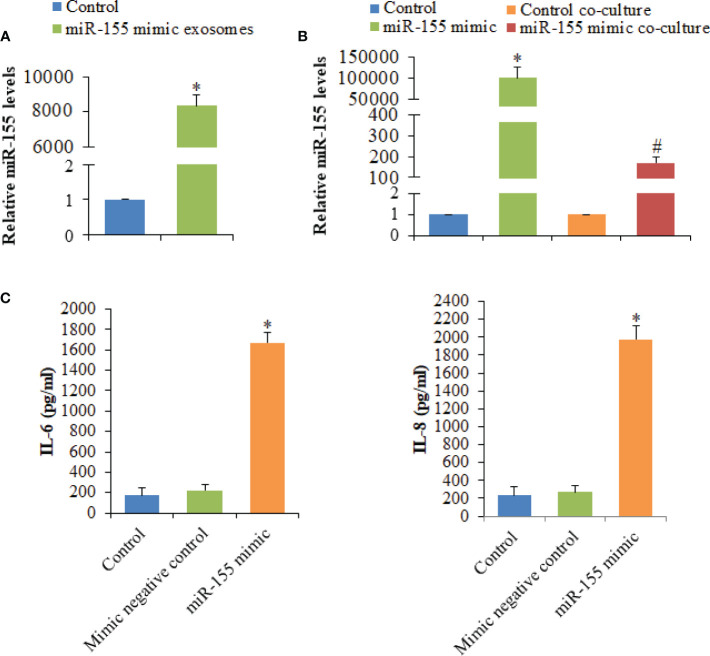
Analysis of the expression levels of miR-155 and its effect on the production of cytokines secreted by macrophages. **(A)** The expression levels of miR-155 in exosomes derived from cardiomyocytes transfected with miR-155 mimic or mimic control. **(B)** The expression levels of miR-155 in macrophages transfected with miR-155 mimic or mimic control and macrophages co-cultured with exosomes derived from cardiomyocytes transfected with miR-155 mimic or mimic control. **(C)** The effects of miR-155 on the production of cytokines. Values are represented as mean ± SD from three independent determinations. *p < 0.05, *vs* control group. ^#^p < 0.05, *vs* control co-culture group.

Among seven significantly differentially expressed miRNAs, miR-155 had the highest expression level in HC-Exo. Also, miR-155 has been identified as the inflammation-associated miRNA (12). Therefore, miR-155 was selected for further investigation. Exosomes were isolated from cardiomyocytes transfected with miR-155 mimic or mimic control. The results showed that miR-155 transfection drastically increased the miR-155 level of exosomes (**Figure 6A**). Additionally, the expression level of miR-155 in macrophages was strongly increased after co-cultured with exosomes derived from cardiomyocytes overexpressing miR-155, confirming the exosome-mediated miR-155 transfection (**Figure 6B**). To better understand the function of miR-155 in the regulation of cytokine production, we determined IL-6 and IL-8 levels of macrophages after the transfection of miR-155 mimic into RAW264.7 cells. We observed that the overexpression of miR-155 in macrophages promotes the production of IL-6 and IL-8, indicating the crucial role of miR-155 in the initiation of inflammation in macrophages (**Figure 6C**).

### Exosomes Interfere with the MAPK Signaling Pathway *via* miR-155

**Figure 7:**
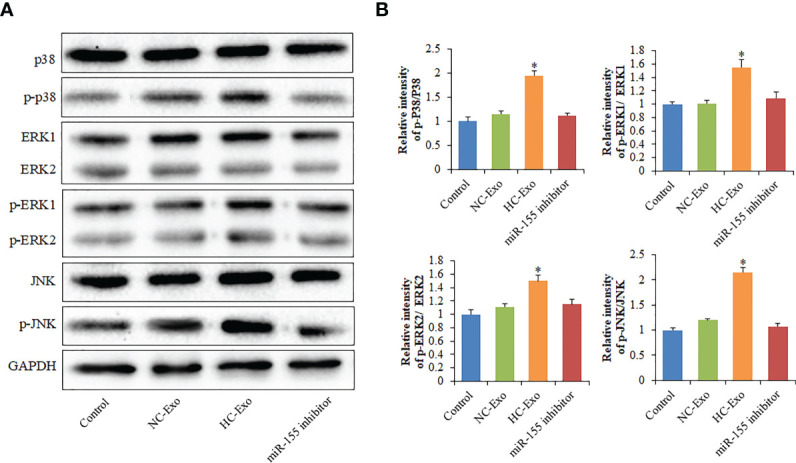
The effects of miR-155 on the MAPK signaling pathway. **(A)** Western blot protein band diagram. **(B)** Densitometry analysis of p38, ERK1/2, and JNK levels, respectively. Values are represented as mean ± SD from three independent determinations. *p < 0.05, *vs* control group.

**Figure 8:**
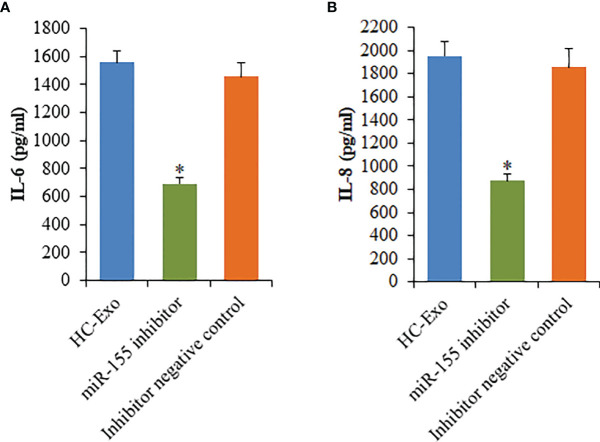
Effects of miR-155 inhibitor on the production of cytokines mediated by cardiomyocyte-derived exosomes. **(A)** IL-6. **(B)** IL-8. Values are represented as mean ± SD from three independent determinations. *p < 0.05, vs HC-Exo group.

To investigate the participation of the MAPK signaling pathway, RAW264.7 macrophages were treated with exosomes. The phosphorylation of c-Jun N-terminal kinase (JNK), extracellular signal-regulated kinase (ERK), and p38 proteins was determined. The phosphorylation of p38, ERK1/2, and JNK was significantly elevated following the treatment with HC-Exo (**Figure 7**). We found that inhibition of miR-155 expression could sufficiently attenuate the phosphorylation of p38, JNK, and ERK in response to exosomes stimulation (**Figure 7**). Moreover, we further observed that miR-155 inhibitor significantly inhibited the production of IL-6 and IL-8 in macrophages (**Figure 8**). These results suggest that exosomes are involved in regulating the MAPK signaling pathway *via* miR-155.

## Discussion

Exosomes are emerging as novel cell-cell communication mediators and play an important role in the regulation of the inflammatory immune microenvironment, which has a profound effect on the development of cardiac hypertrophy (13). The generation and release of exosomes derived from cardiomyocytes have been widely reported (14). Previous studies demonstrated that exosomes from cardiomyocytes play pivotal roles in intercellular communication through the transfer of information between different cell types, including cardiomyocytes, fibroblasts, epicardial cells, bone marrow-derived mesenchymal stem cells, and monocytes (13, 15). Extensive evidence has also documented that activated macrophages secrete mir-155-enriched exosomes, which act as a paracrine regulator for fibroblast inflammation in the injured heart (16). However, little information is available on the crosstalk between cardiomyocytes and macrophages through the exosomes derived from cardiomyocytes. Also, the underlying mechanisms of cardiomyocyte-macrophage communication in the pro-inflammatory cardiac microenvironment that are regulated by exosomes remain unclear.

In this study, we investigated potential crosstalk between cardiomyocytes and macrophages through the cardiomyocyte-derived exosomes. Notably, Ang II-induced hypertrophic cardiomyocytes increase the secretion of exosomes. This is consistent with the findings of the previous study (17). Exosomes are small membrane-bound vesicles secreted by most cell types and could mediate intercellular signaling by shuttling biological macromolecules such as nucleic acids, proteins, and lipids (18). In this report, we found that exosomes isolated from the culture media of HCs trigger the secretion of inflammatory cytokines. We further found that the release of inflammatory cytokines induced by HCs-derived exosomes was prevented by the down-regulation of AGO2. These results indicated that cardiomyocyte-derived exosomes packaged the non-coding RNA-based messages into macrophages and were involved in inflammatory processes in the recipient cells by regulating gene expression.

Many studies demonstrated that exosomes derived from cardiomyocytes were enriched in certain miRNA contents (19). Our analysis revealed 635 differentially expressed miRNAs in exosomes released from hypertrophic cardiomyocytes compared with those in normal cells, and seven miRNAs were identified as significant expression. The present results also illustrated that miR-155 was highly expressed in HCs-derived exosomes. Also, miR-155 can promote the production of cytokines. Extensive evidence has documented that miR-155 is a known regulator of inflammation (20), which is in line with our present results. To investigate the contribution of exosomes in the transfer of miR-155, we overexpressed miR155 in cardiomyocytes which led to the high miR-155 level of exosomes. When we cultivated these exosomes with macrophages, the level of miR-155 was increased in recipient cells. Our results are in agreement with previous studies reporting the exosome-mediated delivery of miR-155 (21).

The present results also provide evidence that that miR-155 activated the MAPK signaling pathway. It was previously reported that miR-155 was involved in the mediation of the MAPK signaling pathway (22). Moreover, miR-155 played a critical role in regulating inflammatory responses through the MAPK signaling pathway (23). Therefore, the present findings of intercorrelation between miR-155 and the MAPK signaling pathway in the regulation of inflammatory processes are consistent with a range of previous results in other systems.

### Limitations of the Study

We have shown that treatment with HCs-derived exosomes leads to inflammation in macrophages *via* miR-155 mediated MAPK signaling pathway. However, several limitations of this study must be noted. On the one hand, this *in vitro* study does not allow us to detect the inflammatory microenvironmental changes induced by HCs-derived exosomes in hypertrophic myocardium, and the assessment of the effects of HCs-derived exosomes on the pro-inflammatory response *in vivo* will be needed. On the other hand, the effect of the MAPK signaling pathway on the production of pro-inflammatory cytokines by immune cells should be explored through treatments with MAPK pathway inhibitors or activators.

## Conclusions

In conclusion, we indicated that exosomes derived from hypertrophic cardiomyocytes were able to activate the miR-155 mediated MAPK pathway, which ultimately induces inflammation in macrophages. The results of the present study may have the potential to provide significant improvements in the effectiveness of the diagnosis and treatment of cardiac hypertrophy.

## Data Availability Statement

The RNA-seq data has been uploaded to NCBI, with the assigned GEO accession number GSE158509.

## Ethics Statement

The animal study was reviewed and approved by Animal Use and Care Committee, Baotou Medical College.

## Author Contributions

HY, LQ, YP, and WB performed the experiments. HY and LQ planned the experiment and analyzed the data. ZW wrote the manuscript. All authors contributed to the article and approved the submitted version.

## Funding

The present study was supported by the National Natural Science Foundation of China (grant nos., 81760056, 82060084, and 81660048), the Natural Science Foundation of Inner Mongolia Autonomous Region of China (grant no., 2019MS08202).

## Conflict of Interest

The authors declare that the research was conducted in the absence of any commercial or financial relationships that could be construed as a potential conflict of interest.
