# Supplementary material for: Exosomes Derived From Hypertrophic Cardiomyocytes Induce Inflammation in Macrophages via miR-155 Mediated MAPK Pathway
Source: Front Immunol. 2021 Feb 3;11:606045. doi: 10.3389/fimmu.2020.606045 (PMC7886800; doi:10.3389/fimmu.2020.606045)
Supplement: Supplementary file 1 [file DataSheet_1.docx]

**Support Information**

**Exosomes derived from hypertrophic cardiomyocytes induce inflammation in macrophages via miR-155 mediated MAPK pathway**

Hui Yu, Lei Qin, Yunzhi Peng, Wenhui Bai, Zhanli Wang*


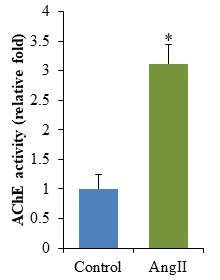


**Figure S1**. AChE activity of exosomes derived from control and Ang II-treated cardiomyocytes, respectively. *p<0.05, *vs* control group.


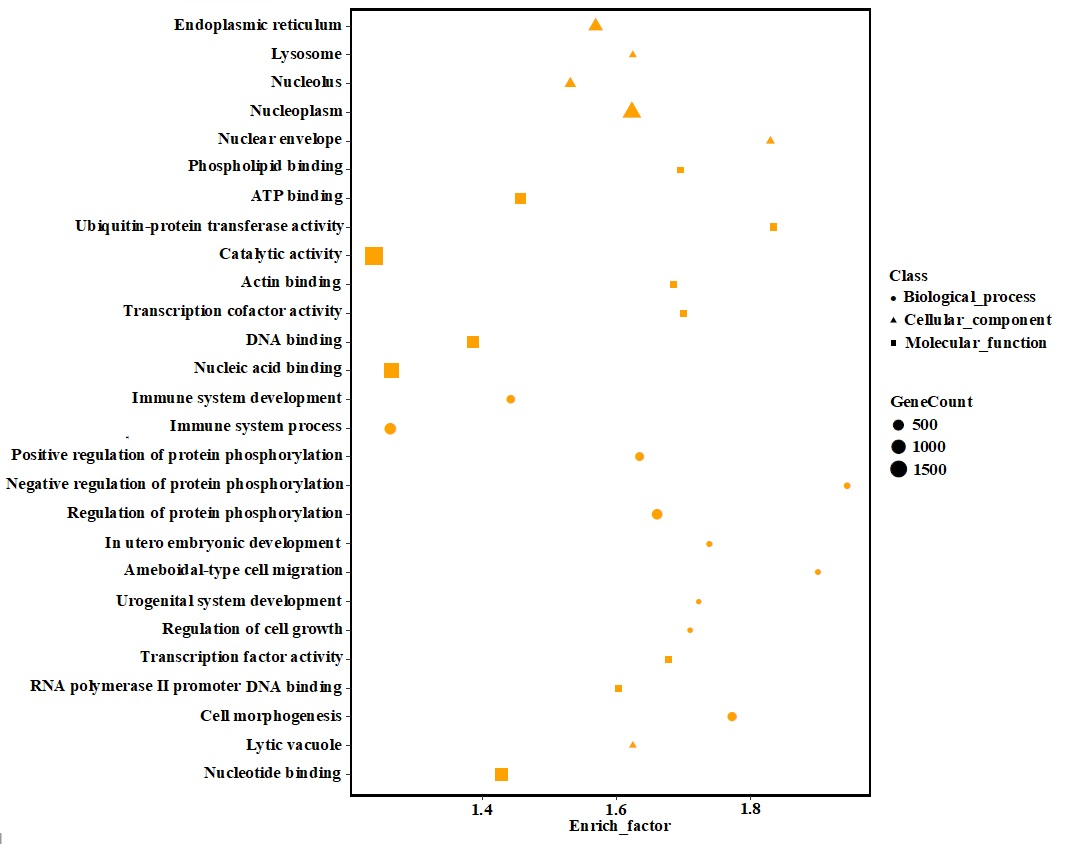


Figure S2. GO annotation for target genes of the differentially expressed miRNAs.
